# Assessing the Impacts of Dairy Farm Antimicrobial Use on the Bovine Fecal Microbiome

**DOI:** 10.3390/ani15121735

**Published:** 2025-06-12

**Authors:** Andrew J. Steinberger, Juliana Leite de Campos, Ashley E. Kates, Tony L. Goldberg, Pamela L. Ruegg, Nasia Safdar, Ajay K. Sethi, John M. Shutske, Garret Suen

**Affiliations:** 1 Department of Bacteriology, University of Wisconsin, Madison, WI 53706, USA; asteinberger@wisc.edu; 2 Microbiology Doctoral Training Program, University of Wisconsin, Madison, WI 53706, USA; 3 Department of Animal Science, Michigan State University, East Lansing, MI 48824, USA; leitedec@msu.edu; 4 Department of Medicine, University of Wisconsin, Madison, WI 53705, USA; akates@medicine.wisc.edu (A.E.K.); ns2@medicine.wisc.edu (N.S.); 5 Department of Pathobiological Sciences, University of Wisconsin, Madison, WI 53706, USA; tony.goldberg@wisc.edu; 6 Department of Large Animal Clinical Sciences, College of Veterinary Medicine, Michigan State University, East Lansing, MI 48824, USA; plruegg@msu.edu; 7 Department of Population Health Sciences, University of Wisconsin, Madison, WI 53726, USA; ajay.sethi@wisc.edu; 8 Department of Biological Systems Engineering, University of Wisconsin, Madison, WI 53706, USA; john.shutske@wisc.edu

**Keywords:** antimicrobial use (AMU), amplicon sequencing, dairy cattle subpopulations, fecal bacterial community structure, AMU quantification metrics

## Abstract

Rising rates of antimicrobial-resistant infections have prompted increased scrutiny on antimicrobial use (AMU) in livestock agriculture. Dairy farms primarily use antimicrobials to maintain animal health and welfare by treating and preventing infectious diseases. However, the impact of dairy farm AMU practices on the cattle fecal microbiome remains largely unclear, partly due to difficulties in quantifying AMU. This study leveraged quantitative AMU data from 40 large commercial dairy farms to identify farms with low (n = 4) and high (n = 4) AMU. Using 16S rRNA gene amplicon sequencing, we compared the fecal bacterial communities of dairy calves and cows (healthy, cull, sick) by both AMU designation (high/low) and by individual farm AMU, summarized by animal defined daily dose (DDD) and mg/kg. We found significant differences in beta-diversity between cattle from high- and low-AMU groups using either method and found that *Corynebacterium* and *Clostridium* abundances increased with farm AMU. Additionally, we found fecal bacterial communities differed across farms within high- and low-AMU groupings, highlighting the need to account for farm-to-farm variation when assessing AMU impacts. These findings suggest that dairy farm AMU influences the fecal microbiome and identifies specific taxa that warrant further investigation as potential reservoirs for antimicrobial resistance genes.

## Simple Summary

The growing issue of antimicrobial-resistant infections has led to increased scrutiny on how antimicrobials are used on dairy farms. Dairy farms use antimicrobials to keep animals healthy by treating and preventing diseases. However, the impact of these practices on the bacteria in cattle feces is not well understood. This study leveraged antimicrobial-use (AMU) data from 40 large dairy farms to identify and compare 4 farms with low and 4 farms with high AMU. By examining the bacteria in the feces of dairy calves and cows from those farms, this study found significant differences in bacterial communities from farms with high and low AMU. Specifically, bacteria such as *Corynebacterium* and *Clostridium* were more prevalent on farms with higher AMU. These findings suggest that AMU on dairy farms may affect the cattle fecal microbiome and highlight specific bacteria that may need further investigation as potential carriers of antimicrobial resistance. Understanding these impacts is crucial for developing strategies to combat antimicrobial resistant pathogens and improve animal health.

## 1. Introduction

With rising rates of antimicrobial-resistant infections, renewed emphasis is being placed on understanding the role of animal agriculture in resistance emergence and dispersal in pathogens [1,2,3]. Farms are known sources of pathogens, with animal feces considered fomites for dispersal both across the farm and into the surrounding environments [4,5]. Combined with close interactions between humans and the agricultural environment, these reservoirs are considered a significant risk to both human and animal health [6]. Assessing the role of farm antimicrobial use (AMU) and its impact on the fecal microbiome of cattle would allow for a better understanding of pathogen dynamics in the farm environment.

Given the challenges in quantifying AMU on dairy farms, it is unsurprising that our understanding of its impact on the bovine fecal microbiome is not well understood relative to other livestock species. Prior work connecting herd-level antimicrobial use to the fecal microbiome of dairy cattle has been largely limited to comparisons of antimicrobial resistance of culturally recovered pathogens, often from organic (with no or limited antimicrobial use) and conventional (antimicrobial using) farms. Results from these studies have been variable, with some reporting increased resistance in isolates recovered from feces on conventional farms [7,8,9] and others finding similar levels of resistance regardless of farming practices [10,11].

More recent work has leveraged next-generation sequencing technologies, including 16S rRNA gene amplicon and shotgun metagenomic sequencing, to further compare the whole fecal microbiomes and resistomes of cattle from organic and conventional dairy farms. These efforts have generally yielded similar findings to the bacterial isolate studies, finding differences in the resistomes, but not the microbiomes, of feces from dairy cattle raised conventionally and organically [12,13,14].

Several studies have explored the acute impacts of individual antimicrobial treatments or exposures on the dairy bovine fecal microbiome and resistome, with many reports finding either transient shifts in the microbiome and ARG presence that return to baseline soon after the end of a given treatment [15,16,17], or no change in the fecal microbiome or resistome [18,19]. However, recent work by Vasco and colleagues [20] found that a single intramammary treatment of ceftiofur, a 3rd generation cephalosporin commonly used on dairy farms at dry-off, resulted in an increased abundance of specific bacterial taxa and extended-spectrum beta-lactamase (ESBL) encoding genes in the feces of treated animals throughout their 9-week study. However, they found no lasting changes to the broader fecal microbiome of treated cattle in response to treatment and found significant animal-to-animal variation in fecal shedding of cultivable bacteria within the herd.

Less work has been performed to evaluate how AMU practices on conventional dairy farms impact cattle fecal microbiomes, even though conventional dairying is practiced across the majority of US dairy farms [21]. A pilot study by Firth et al. [22] examined antimicrobial resistant bacterial isolates from animal housing areas on 25 high- and 25 low-AMU dairy farms in Austria and found no difference in isolate recovery of ESBL *Escherichia coli* between groups. More recently, an evaluation of AMU impacts on the microbiome and resistome of beef cattle by Doster et al. [23] found that antimicrobial exposures do not strongly affect the fecal microbiome nor its resistome when compared to other factors that were quantified.

Due to inconsistent results, some recent studies have suggested that factors external to farm AMU can impact ARG selection within the microbiomes on dairy farms, including farm-to-farm variation in environment, location, diet, or non-antimicrobial management practices and treatments [14,24]. However, most studies to date have lacked access to conventional dairy farms with robust AMU data. Here, we sought to overcome this limitation by leveraging prior work from our group that quantified the AMU of 40 conventional dairy farms in Wisconsin, the USA [25]. Guided by these AMU measures, we quantified and compared the fecal bacterial communities of cattle from select farms having either high or low AMU to determine whether farm AMU is associated with differences in the fecal microbiome composition. The goal of this study was to better understand whether the bacterial community compositions of cattle feces are impacted by farm AMU practices in the dairy farm environment, thereby facilitating the future study of pathogen dynamics and potential resistance emergence on dairy farms.

## 2. Materials and Methods

Farms enrolled in this study were selected from 40 commercial dairy farms that were the focus of a prior study by our group to quantify the AMU of conventional dairy farms in Wisconsin, USA [25]. That study collected and summarized 1 year of antimicrobial usage data for farms meeting the following enrollment criteria: farms had to be “large”, having ≥250 lactating dairy cows when initially contacted, had to have used antimicrobials to treat or prevent at least 1 disease event in the previous year, and had to have kept digital records of their antimicrobial treatments. Usage data were collected in the fall of 2017 and covered AMU for the 365 days prior to the date of data collection. Additionally, AMU was quantified using 2 standardized metrics: (1) number of animal defined daily doses (DDD) administered, normalized per 1000 animal-days, and (2) mg of antimicrobial administered per kg of animal weight (mg/kg), as previously described [26,27,28,29]. All AMU data reported in this study represent a subset of the data from our previous work [25]. Here, we aimed to re-enroll 4 farms with high AMU and 4 farms with low AMU. To select these farms, the 40 farms initially surveyed were sorted by farm AMU (using the DDD metric) and farms with the highest and lowest AMU were sequentially contacted via letter and/or phone for re-enrollment until 4 high and 4 low farms were successfully enrolled. Altogether, 6 high-AMU farms and 11 low-AMU farms were re-contacted for this study.

**Figure 1:**
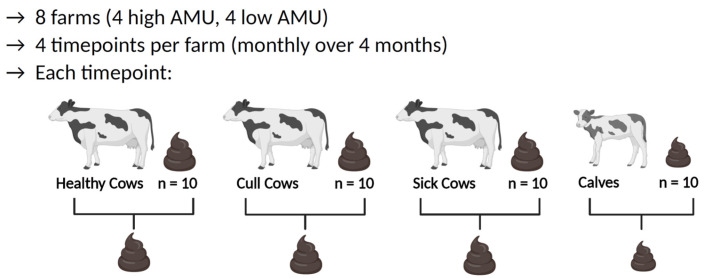
Sampling methods. Each of the 8 enrolled farms, 4 having high AMU and 4 having low AMU, were visited 4 times. At each sampling visit, fecal samples were collected from 10 cattle from each of the 4 cattle groups (preweaned calves, healthy lactating cows, sick cows, and cull cows) and pooled into a single composite sample prior to DNA extraction and sequencing. This image was created with https://www.biorender.com/.

Bovine fecal sampling was performed between January and October 2020 using a schedule and sampling method adapted from prior studies [30,31,32], with the exception of a period from mid-March through mid-June when sampling was paused due to the COVID-19 pandemic. Briefly, each farm was visited 4 times at monthly intervals. At each visit, fecal samples were collected directly, as available, from 10 individual animals from each of 4 different cattle groups: preweaned heifer calves, cows to be culled, healthy lactating cows, and cows in a designated sick pen (Figure 1).

Individual animals were identified for sampling using a systematic random sampling approach. Briefly, the total number of animals in the group to be sampled (*N*) were divided by the number of samples to be collected from that group (*n*) to determine a sampling interval *k* (*N*/*n* = *k*). Every *k*th animal in that group, equally distributed across pens and barns, was sampled. For cull cows, this was performed using a list of ear tag numbers of all cows to be culled and their pen locations provided by the farm manager at the start of each visit. Cattle groups were sampled in the order of calves to healthy cows and cull cows and then to sick cows for each visit. No effort was made to avoid collecting samples from the same individual animal on subsequent visits or to avoid sick calves when conducting systematic random sampling. Once animals were identified, ~40 g of fecal material was collected directly from the rectum of each animal using either shoulder-length gloves (Nasco, Fort Atkinson, WI, USA) for cows or nitrile gloves (Ansell, Iselin, NJ, USA) for preweaned calves. Separate clean gloves were used for each sample. If it was not possible to obtain at least 40 g of fecal material, as much fecal material as possible was collected. Samples were placed into sterile 50 mL conical tubes (Falcon, Fisher Bioscience, Waltham, MA, USA) and immediately stored on wet ice before being transported to the laboratory and stored at −80 °C. To avoid cross contamination between farms, stocks of supplies (glove boxes, falcon tubes, etc.) were kept separate for each farm, all clothing was laundered, and all boots and equipment were washed and sanitized after each visit.

In the laboratory, individual fecal samples collected from each cattle group per visit were thawed and combined at equal mass (~10g where available) into sterile Whirl-Pak bags (Nasco, Oakbrook Terrace, IL, USA) and homogenized by hand squeezing until thoroughly visibly mixed (1–2 min). A total of ~2 g of the resultant composite sample was then transferred into sterile 2 mL screw-cap tubes (VWR/Avantor, Radnor, PA, USA) and stored at −80 °C until DNA extraction. After pooling, all samples were assigned randomized labels and remained randomized until sequence analysis to prevent bias and batch effects during processing.

Randomized composite samples were thawed, and DNA was extracted directly from 222–253 mg of sample using the QIAamp PowerFecal Pro DNA extraction kit (Qiagen, Germantown, MD, USA) according to manufacturer’s instruction. One tube per batch was reserved as a negative control and was treated identically to all samples. Resultant DNA samples were quantified using the Qubit Broad-Range assay kit (Thermo Fisher Scientific, Waltham, MA, USA) on a Synergy 2 Multi-Mode plate reader (BioTek, Winooski, VT, USA). The V4 hypervariable region of the bacterial 16S rRNA gene was amplified by polymerase chain reaction (PCR) using barcoded, 1-step sequencing primers as described in Kozich et al. [33]. PCR reactions were performed using 25 ng template DNA, 0.5 µL forward and reverse primers at 10 mM, 12.5 µL KAPA 2×HiFi Master Mix (Roche, Rotkreuz, Switzerland), and nuclease free water (IDT, Coralville, IA) to a total volume of 25 µL. Water was used in place of template DNA for PCR negative controls, which were processed identically to fecal samples.

PCR conditions were as follows: 95 °C for 3 min, 25 cycles of 95 °C for 30 s, 55 °C for 30 s, and 72 °C for 30 s, followed by a final extension at 72 °C for 5 min. PCR products were run on a 1% (*w*/*v*) low-melt agarose gel, desired PCR products (~380 bp) were excised, recovered using a 96-well Zymoclean Gel DNA Recovery Kit (Zymo Research, Irvine, CA, USA), and quantified using a Qubit High-Sensitivity assay kit (Thermo Fisher Scientific, Waltham, MA, USA) and a Synergy plate reader. DNA samples were equimolarly pooled to 4nM and sequenced on an Illumina MiSeq (Illumina, Inc., San Diego, CA, USA) using a 500-cycle MiSeq v2 sequencing kit with custom sequencing primers [33].

Paired-end reads were demultiplexed on the Illumina MiSeq, de-randomized, then processed using dada2 v1.28 [34] in R (v. 4.3.1) [35] following standard protocols. Briefly, raw paired-end reads were trimmed (forward to 240 bp, reverse to 220 bp), and reads having ambiguous base calls, >2 expected errors, and those aligned to PhiX were removed. Error rates were learned, sample inference was run, and resultant reads were merged into amplicon sequence variants (ASVs). ASVs with lengths less than 250 bp or greater than 256 bp were removed, and chimeric sequences were identified and removed using default settings. Remaining ASVs were then taxonomically classified against the SILVA taxonomic database (v138.1) [36] with an 80% minimum confidence cut-off.

Quality controlled sequences were imported into phyloseq v1.44.0 [37] for further processing. Contaminant ASVs were predicted and removed using decontam v1.19.0 [34] with method = prevalence. Additionally, ASVs classified as Archaea, chloroplast, or mitochondria were also removed. Samples were then rarefied to a depth of 10,334 sequences per sample, and alpha diversity metrics, Chao’s Richness, and Shannon’s Diversity Index were calculated in phyloseq. The sequences supporting this study are available in the NCBI’s Sequence Read Archive under BioProject number PRJNA1093066 [https://www.ncbi.nlm.nih.gov/bioproject/PRJNA1093066 (accessed on 5 June 2025)].

Statistical analyses were performed in R. Bacterial communities were primarily assessed by farm AMU category (high or low) or by farm AMU as a continuous value for each AMU metric (DDD and mg/kg.) For continuous AMU, the AMU value assigned to a sample was specific to whether the sample was collected from calves or adult cows. Alpha diversity was assessed using both Chao’s Richness estimate and Shannon’s Diversity Index, a measure of richness and evenness. Normality was evaluated using the Shapiro–Wilk test. For comparison between high- and low-AMU farms, significance was determined using an ANOVA or the Kruskal–Wallis (KW) test depending on normality, with cattle group and farm included as fixed effects where appropriate to account for their potential impacts external to farm AMU practices. Post hoc pairwise comparisons were similarly performed using Tukey’s HSD or the Wilcoxon Rank Sum test in the case of significance (*p* < 0.05). When evaluating alpha diversity by farm AMU as a continuous metric (using DDD or mg/kg), a general linear model was used with cattle group and farm included as fixed effects where appropriate. Significance was determined using the Wald test, the default test for stats::summary in R [35]. Longitudinal sampling effects on alpha diversity metrics were evaluated using a linear mixed effects model with farm included as a random effect, and significance was again determined using the Wald test.

Beta diversity was evaluated using Bray–Curtis dissimilarity, calculated using the R package vegan v2.6-4 [38], and both unweighted and weighted UniFrac metrics, calculated using a GTR + G + I (generalized time-reversible with gamma rate variation) maximum likelihood tree of ASVs from a neighbor-joining tree, with the R packages phangorn (v2.11.1) [39], ape::NJ (v5.7-1) [40], and phyloseq::UniFrac [37,41]. Beta-dispersion was tested (vegan::betadisper), and permutational-multivariate-ANOVA (PERMANOVA) (vegan::adonis2) or analysis of similarities (ANOSIM) (vegan::anosim) were used to assess significance depending on dispersion for all beta-diversity analyses with permutations = 10,000. Cattle group and farm were included as fixed effects for AMU comparisons where appropriate.

ASVs driving community differences by farm AMU were identified using ANCOM-BC2 v2.2.2 [42] on non-rarefied counts using default settings, and an FDR-adjusted *p*-value cutoff of *p* < 0.05 was used to determine significance. Calf and cow samples were evaluated separately. Calf samples were evaluated with the fixed model “AMU” and cows with “AMU + type”, with type being cattle groups to account for variability between cow groups (healthy, cull, sick). Farm-to-farm variability was initially included in the models, but there was too much multicollinearity for ANCOMBC-2 to attempt taxa identification and was thus removed.

## 3. Results

### 3.1. Sampling and Sequencing Summary

In this study, 1145 animals were sampled during 32 visits to the eight enrolled farms, resulting in 116 composite fecal samples (Figure 1). Of composite samples, 24 were from calves, 32 from healthy lactating cows, 32 from cull cows, and 28 from sick cows. Fecal samples collected from low-AMU farms generated 52 composite samples (calf: 8, healthy: 16, cull: 16, sick: 12) while high-AMU farms generated 64 composite samples (calf: 16, healthy: 16, cull: 16, sick: 16). Discrepancies in sample counts result from two low-AMU farms (L1 and L4) not rearing or treating calves on-site, preventing sample collection, and one low-AMU farm (L3), which did not maintain a sick pen nor designate cows for antimicrobial treatment, preventing sample collection. Additionally, the sick pen of farm L4 contained fewer than 10 cows at each visit, so the composite sick samples for these visits comprised 3–9 individual fecal samples rather than 10 as in all other samples. A list of all samples and their associated metadata are available in Tables S1 and S2.

In total, 4,968,410 raw sequences were generated, 4,961,946 from the 116 fecal samples and 6464 from the 12 DNA extraction and PCR controls. After cleanup, 3,032,366 sequences remained (samples: 3,032,262, controls: 104), resulting in 4372 unique ASVs, representing 17 phyla, 102 families, and 267 genera. After rarefaction to 10,334 sequences per sample, total counts included 1,198,744 reads and 4175 unique ASVs, constituting 17 phyla, 94 families, and 254 unique genera from the 116 fecal samples. All negative controls were removed during sample rarefaction, as their final sequence counts were <30 sequences per sample.

### 3.2. Farm Antimicrobial Use

**Figure 2:**
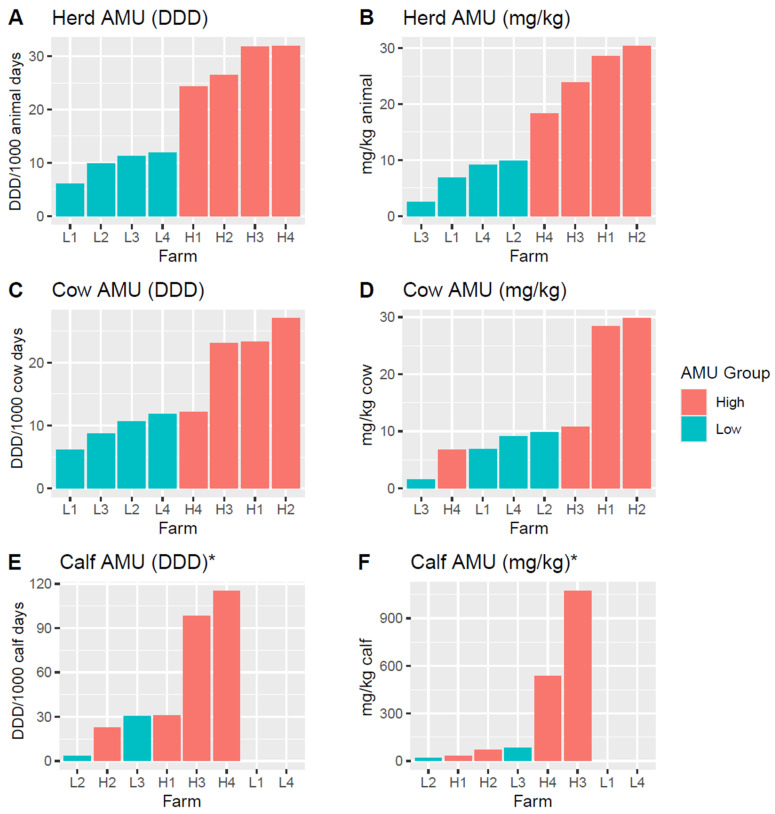
Antimicrobial use of enrolled farms. Bar plots detailing the antimicrobial use of each enrolled farm with bars colored according to an initial designation as a “high” or “low” AMU farm. Plots (**A**,**C**,**E**) describe farm AMU using the animal defined daily dose (DDD) metric, normalized per 1000 animal days, with plot (**A**) showing AMU across all cattle on each farm and plots (**C**,**E**) showing the AMU subset to usage only on adult animals (**C**) or preweaned calves (**E**). Plots (**B**,**D**,**F**) describe farm AMU using the mg/kg metric. Bars are ordered left-to-right by increasing AMU. There is no calf AMU data for farms L1 and L4, as they did not raise preweaned calves on-site. * Note that although calf AMU appears greater than cow AMU, it represents only a small proportion of herd AMU since preweaned calves are only a small fraction of the total population, are only housed for ~6–8 weeks until weaning, and weigh much less than adult cattle.

AMU for enrolled farms was assessed using data from Leite de Campos et al. [25], which were summarized using the standardized metrics DDD and mg/kg as previously described [25,26,27,28,29]. Low-AMU farms averaged 9.76 ± 1.29 DDD/1000-animal days and 7.13 ± 1.66 mg/kg, and high-AMU farms averaged 28.6 ± 1.94 DDD/1000-animal days and 25.26 ± 2.69 mg/kg. Farms enrolled into our study from the “high” and “low” categories retained their groupings when summed by either metric, with only the rank of individual farms within their category differing between metrics (Figure 2A,B).

However, when AMU was examined separately for only preweaned calves or for only cows, the initial high/low classifications no longer held. For cow-specific AMU, high-AMU farm H4 had an AMU more similar to the low-AMU farms using either metric, while high-AMU farm H3 also had more similar usage to low-AMU farms, but only for mg/kg (Figure 2C,D). Similarly, when examining calf-specific AMU, high-AMU farms H1 and H2 had calf AMU more similar to those of low-AMU farms when using either metric (Figure 2E,F). Given this, cow- and calf-specific AMU values were used when analyzing AMU impacts on cattle sub-groups. Additionally, although calf AMU appears numerically greater than AMU for cows, calf AMU contributes a smaller proportion of total herd AMU compared to cows, as preweaned calves are fewer in number relative to the total herd, are only housed for ~6–8 weeks until weaning, and are physically smaller than cows.

### 3.3. Bacterial Taxonomic Compositions

Taxonomic compositions of calf and adult cattle fecal samples were considered separately due to expected differences in composition. Calf fecal samples across all farms were primarily dominated by three phyla: the Firmicutes (68 ± 2%), Bacteroidota (19 ± 2.5%), and Actinobacteriota (11 ± 1.3%), with Proteobacteria (1.1 ± 0.2%), Spirochaeota (0.1 ± 0.04%), and Desulfobacterota (0.1 ± 0.04%) being the next most abundant. At the genus level, the five most abundant genera were the *Faecalibacterium* (11 ± 1.8%), *Blautia* (11 ± 2.3%), *Bifidobacterium* (8.8 ± 1.9%), *Bacteroides* (7.3 ± 1.6%), and *Provotella_9* (6.1 ± 2.7%) (Figure S1A,B).

Bacterial communities of adult cattle across all farms were primarily composed of the phyla Firmicutes (74 ± 0.4%) and Bacteroidota (21 ± 0.4%) and to a lesser extent by Actinobacteria (2.5 ± 0.3%), Spirochaetes (1.4 ± 0.1%), Verrucomicrobiota (0.3 ± 0.3%), and Proteobacteria (0.2 ± 0.03%). The most abundant genera in cow fecal samples were the *Ruminococcaceae UCG-005* (20 ± 0.6%), *Romboustia* (10 ± 0.5%), *Rikenellaceae* RC9 (7.6 ± 0.3%), *Christensenellaceae R-7* (6.3 ± 0.3%), and *Prevotellaceae UCG-003* (4.2 ± 0.2%) (Figure S1C,D).

### 3.4. Longitudinal Impacts on Bacterial Diversity

Bacterial community diversity was first compared across the four sampling visits with individual farm included as a random effect. No differences in alpha diversity were found between farm visits for either Chao’s richness (*p* = 0.7, Wald) or Shannon’s diversity (*p* = 0.81, Wald). Similarly, for beta diversity, there were no differences between farm visits when determined using Bray–Curtis dissimilarity (*p* = 0.58, PERMANOVA) or unweighted (*p* = 0.89, PERMANOVA) or weighted (*p* = 0.61, PERMANOVA) UniFrac distances. As a result, sampling visits were considered replicates for subsequent analyses.

### 3.5. Farm-to-Farm Variability

Variability of fecal bacterial communities among farms was compared separately for high- and low-AMU farms and separately for calves and cows within each group. A comparison of the microbiome of calves among high-AMU farms revealed differences in richness among farms (Chao: *p* = 0.03, ANOVA) but no differences in diversity among farms (Shannon: *p* = 0.11, ANOVA) (Figure S2A,B). Beta diversity also differed among farms for calves on high-AMU farms using Bray–Curtis dissimilarity (*p* = 9.9 × 10^−5^, PERMANOVA) (Figure S2C) and both UniFrac metrics (*p* < 0.05, PERMANOVA). Similar results were found when comparing samples from cows on high-AMU farms for both alpha (Chao: *p* = 0.02, Shannon: *p* = 0.06, ANOVA) (Figure S2D,E) and beta diversity (Bray–Curtis: *p* = 9.9 × 10^−5^, PERMANOVA, Unweighted UniFrac: *p* = 9.9 × 10^−5^, anosim, Weighted UniFrac: *p* = 9.9 × 10^−5^, PERMANOVA) (Figure S2F).

Our analysis of the variability of calf fecal microbiomes between low-AMU farms is limited by a sample size of eight samples from two farms. Alpha diversity did not differ between the two farms (Chao: *p* = 0.12, Shannon: *p* = 0.84, ANOVA) (Figure S3A,B). However, despite the small sample size, bacterial community composition differed by farm when using Bray–Curtis (*p* = 0.03, PERMANOVA) (Figure S3C) and unweighted UniFrac (*p* = 0.03, PERMANOVA) but was only a strong trend when using weighted UniFrac distances (*p* = 0.08, PERMANOVA). The significance of Bray–Curtis but not weighted UniFrac indicates that though the bacterial communities differ, many of the differing bacterial groups are taxonomically similar, though this conclusion should be made cautiously due to the small sample size. Further comparisons of cow fecal microbiome variability across all four low-AMU farms found differences in diversity (Shannon: *p* = 0.02, ANOVA) but not sample richness (Chao: *p* = 0.1, ANOVA) (Figure S3B,C). Bacterial community composition differed (*p* = 9.9 × 10^−5^, PERMANOVA) for Bray–Curtis (Figure S3A) and unweighted and weighted UniFrac metrics. Thus, farm variability should be considered when evaluating AMU effects and will be included in subsequent analyses.

### 3.6. Bacterial Diversity Differs Between High- and Low-AMU Farms

**Figure 3:**
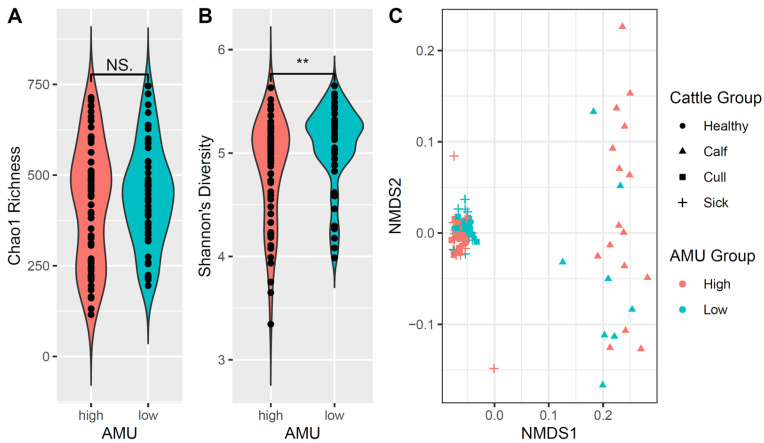
A comparison of bacterial diversity between farms with high and low AMU. Violin plots of Chao’s richness (**A**) and Shannon’s diversity (**B**) for all fecal samples from farms with high and low antimicrobial use (AMU). Plots are colored by farm AMU and comparisons with *p*-values < 0.01 are indicated by **. Plot (**C**) is a non-metric multidimensional scaling (nMDS) plot of the weighted UniFrac distances for the fecal bacterial communities from high- and low-AMU farms. Each dot represents the fecal bacterial community of a single composite fecal sample. Colors indicate farm AMU group and shape indicate the cattle group to which each sample belongs. Stress (**C**) = 0.06. NS = no significant.

Bacterial community diversity was next compared between farms initially identified as having “high” or “low” AMU. For alpha diversity, neither Chao’s richness nor Shannon’s diversity values were normally distributed across our sample set, so the KW test was used. Chao’s richness (*p* = 0.56, KW) did not differ by farm AMU (Figure 3A), but Shannon’s diversity was greater in samples from low-AMU farms than high-AMU farms (*p* = 0.005, KW), seemingly due to fewer low-diversity samples on low-AMU farms (Figure 3B).

Beta diversity was evaluated using Bray–Curtis dissimilarity and weighted and unweighted UniFrac distances. Cattle group and farm were both included as fixed effects as they were found to impact bacterial community composition (Figure S4). A comparison of Bray–Curtis distances found bacterial community composition to differ between cattle feces from farms with high or low AMU (R^2^: 2.8%, *p* = 9.9 × 10^−5^, PERMANOVA). Similar results were found when comparing community composition using weighted (Figure 3C) and unweighted UniFrac distances (R^2^: 1.7%, R^2^: 2.8% resp., *p* = 9.9 × 10^−5^, PERMANOVA). Cattle group and individual farm effects also significantly drove microbiome differences regardless of AMU metric used (*p* < 0.05).

### 3.7. Differentially Abundant Taxa Between High- and Low-AMU Farms

**Figure 4:**
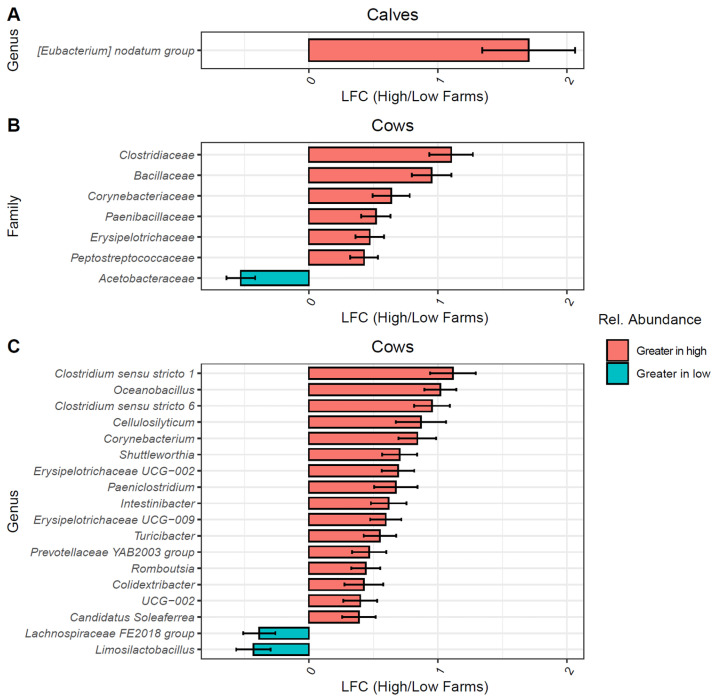
Differentially abundant bacterial taxa between high- and low-antimicrobial-use farms. Bar plots of bacterial genera (**A**,**C**) and families (**B**) found to be differentially abundant between high- and low-antimicrobial-use (AMU) calves (**A**) and all cows (**B**,**C**). Taxonomic groups with FDR-corrected *p*-values > 0.05 were not included. Colors represent log-fold change (LFC) in relative abundance, red for taxa with greater abundances in samples from high-AMU farms, and blue for taxa with greater abundances on low-AMU farms.

Taxonomic groups driving the bacterial community differences observed between high- and low-AMU farms were identified using ANCOMBC-2. Calves and cows were analyzed separately to avoid identifying calf-specific taxa due to uneven calf sampling between groups. For calves, only the genus *Eubacterium* was differentially abundant between calves on high and low farms, being enriched on high-AMU farms (Figure 4A).

For cows, the bacterial families *Clostridiaceae*, *Bacillaceae, Corynebacteriaceae*, *Paenibacillaceae*, *Erysipelotrichaceae*, and *Peptostreptococcaceae* were significantly enriched on high-AMU farms and only *Acetobacteraceae* was enriched on low-AMU farms (Figure 4B). At the genus level, 18 genera were differentially abundant, including 16 enriched on high-AMU farms and 2 on low-AMU farms (Figure 4C). Notable genera enriched in high-AMU farms include two genera of *Clostridium*: *sensu stricto 1* and *6*, *Corynebacterium*, *Cellulosilyticum*, and *Shuttleworthia*. The genera enriched on low-AMU farms were *Limosilactobacillus* and *Lachnospiraceae* group FE2018.

### 3.8. Bacterial Diversity and Broader Antimicrobial Use

We next sought to evaluate if AMU, when treated as a continuous variable for DDD or for mg/kg, resulted in differences in fecal bacterial diversity. Cow or calf AMU values for DDD or mg/kg were assigned to cow and calf samples, respectively, for this analysis. Analyses were performed for all animals (herd) and calves and cows individually.

#### 3.8.1. Herd

For alpha diversity, neither richness nor diversity significantly co-varied with DDD (Chao: *p* = 0.85, Shannon: *p* = 0.56, Wald) or mg/kg (Chao: *p* = 0.71, Shannon: *p* = 0.13, Wald). However, Bray–Curtis and weighted and unweighted UniFrac all significantly covaried with AMU when using either DDD (R^2^: 1.7%, 1.3%, 2.0% resp., *p* < 0.05, ANOVA) or mg/kg (R^2^: 1.8%, 1.2%, 1.9% resp., *p* < 0.05, ANOVA), mirroring the findings of our high/low-AMU comparison.

#### 3.8.2. Calves

The comparison of calf-specific AMU using DDD and calf fecal bacterial communities found both bacterial richness (Chao: *p* < 0.01, Wald) and diversity (Shannon: *p* = 0.01, Wald) to significantly increase with farm AMU. Comparisons of beta diversity also found fecal communities to co-vary with AMU when using Bray–Curtis (R^2^: 5.1%, *p* = 0.04, PERMANOVA), and unweighted UniFrac (R^2^: 6.1%, *p* = 0.01, PERMANOVA) metrics. However, community compositions of weighted UniFrac (R^2^: 2.6%, *p* = 0.51, PERMANOVA) did not covary with AMU, indicating that the bacterial groups that differ with DDD are likely taxonomically similar.

When using the mg/kg metric to evaluate bacterial community differences by AMU, similar results were found for both alpha (Chao: *p* < 0.01, Shannon: *p* = 0.01, Wald) and beta (Bray–Curtis: R^2^: 7.7%, *p* = 0.005, PERMANOVA, Unweighted UniFrac: R^2^: 5.4%, *p* = 0.03, PERMANOVA) diversity with the exception of weighted UniFrac (R^2^: 6.2%, *p* = 0.08, PERMANOVA), which had a lower *p*-value, trending toward significance, and explained a larger proportion of variance than with DDD.

#### 3.8.3. Cows

The comparison of cow-specific AMU and fecal bacterial community revealed no difference in richness (Chao: *p* = 0.29, Wald) or diversity (Shannon: *p* = 0.17, Wald) with DDD. Bacterial community compositions were impacted by DDD AMU when using Bray–Curtis (R^2^: 6%, *p* = 9.9 × 10^−5^, PERMANOVA), unweighted UniFrac (R^2^: 2.1%, *p* = 8 × 10^−4^, PERMANOVA), and weighted UniFrac (R^2^: 11%, *p* = 9.9 × 10^−5^, PERMANOVA). Similar results were found when summarizing AMU using mg/kg for alpha (Chao: *p* = 0.29, Shannon: *p* = 0.17, Wald) and beta (Bray: R^2^: 4%, *p* = 9.9 × 10^−5^, Unweighted UniFrac: R^2^: 1.7%, *p* = 0.007, Weighted UniFrac: R^2^: 5.2%, *p* = 9.9 × 10^−5^, PERMANOVA) diversity metrics of the cow fecal bacterial communities.

### 3.9. Differentially Abundant Taxa by Antimicrobial Usage

**Figure 5:**
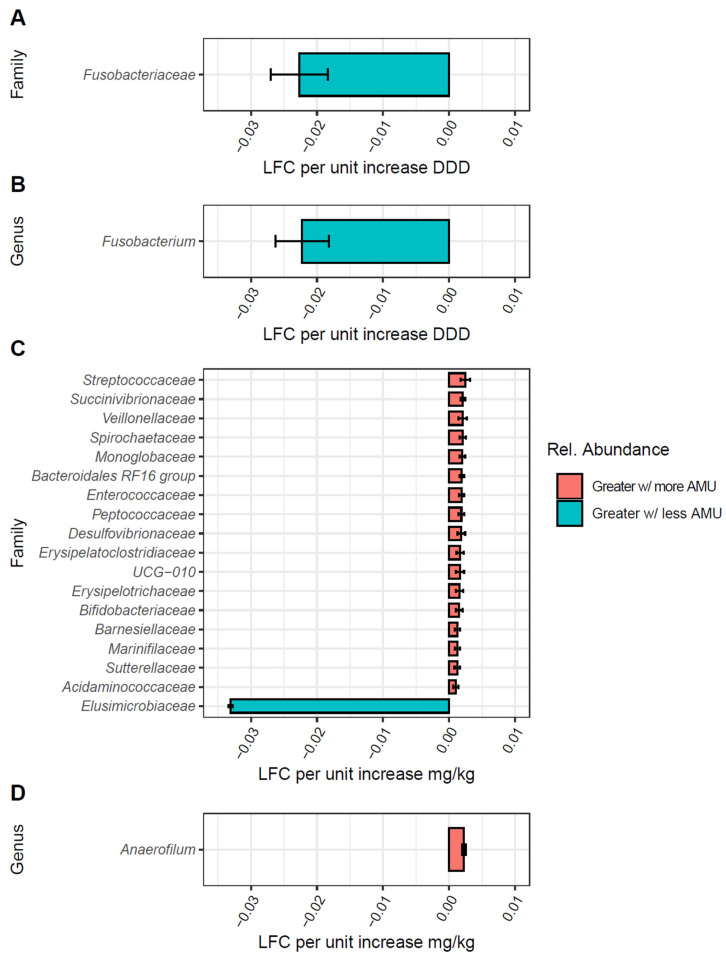
Differentially abundant bacterial taxa in calves by AMU quantified as DDD or mg/kg metrics. Bar plots of bacterial families (**A**,**C**) and genera (**B**,**D**) found to be differentially abundant in calf samples per unit increases or decreases in antimicrobial use (AMU) when using DDD (**A**,**B**) or mg/kg (**C**,**D**). Taxonomic groups with FDR-corrected *p*-values > 0.05 were not included. Colors represent log-fold change in abundance, red for increased log-fold change (LFC) in relative abundance with increasing farm AMU, and blue for increased LFC with decreasing farm AMU.

Given the observed significant changes in bacterial community compositions with both DDD and mg/kg, we used ANCOMBC-2 to identify taxonomic groups driving these differences. For calves, using the DDD metric, we found only the family *Fusobacteriaceae* and the genus *Fusobacterium* to be negatively associated with increasing AMU (Figure 5A,B).

Using mg/kg, 17 bacterial families had significant log-fold changes (LFC) (Figure 5C). Of these, 16 families had positive LFC in calf feces with increasing AMU, with *Streptococcaceae*, *Succinivibrionaceae*, and *Veillonellaceae* having the largest LFC, although all 16 had LFCs of <0.01-fold per unit increase of mg/kg. At the genus level, only the genus *Anaerofilum* had a positive LFC with mg/kg; however, it also had a low LFC in abundance per unit increase on AMU (Figure 5D).

**Figure 6:**
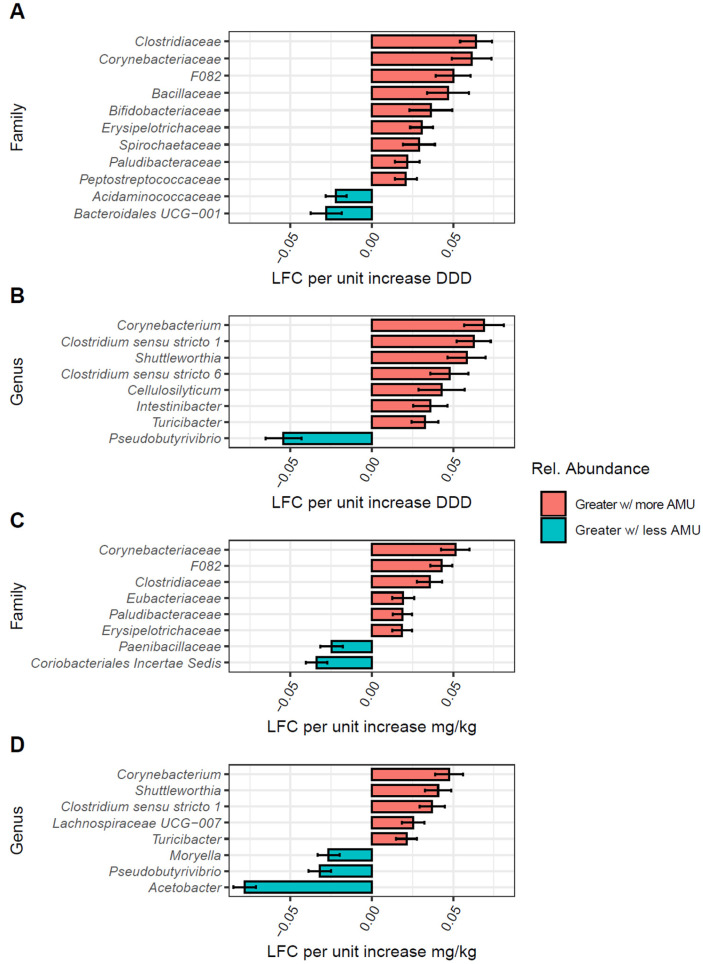
Differentially abundant bacterial taxa in cows by AMU quantified as both DDD and mg/kg metrics. Bar plots of bacterial families (**A**,**C**) and genera (**B**,**D**) found to be differentially abundant in cow samples per unit increases or decreases in antimicrobial use (AMU) when summarized using DDD (**A**,**B**) or mg/kg (**C**,**D**). Taxonomic groups with FDR-corrected *p*-values > 0.05 were not included. Colors represent log-fold change in abundance, red for increased log-fold change (LFC) in relative abundance with increasing farm AMU, and blue for increased LFC with decreasing farm AMU.

For cows, when using the DDD metric, 11 families had significant LFC with AMU, 9 increasing with DDD and 2 decreasing (*Acidaminococcaceae* and *Bacteridales UCG-001*) (Figure 6A). Of these, *Clostridiaceae* and *Corynebacteriaceae* had the highest LFC with DDD, followed by *F082*, *Bacillaceae*, and *Bifidobacteriaceae*. Eight genera were also differentially abundant with *Corynebacterium*; two genera of *Clostridium*: *sensu stricto 1* and *6*, *Cellulosilyticum*, *Shuttleworthia*, *Intestinibacter*, and *Turicibacter* having positive LFC; and only *Psuedobutyrivibrio* having negative LFC with DDD (Figure 6B). Using mg/kg, eight families were differentially abundant with increasing mg/kg: six positively and two negatively associated (Figure 6C).

The results for mg/kg were similar to DDD. We found that *Corynebacteriaceae*, *F082*, *Clostridiaceae*, *Erysipelotrichaceae*, and *Paludibacteraceae* were positively associated with increasing mg/kg, along with *Paludibacteraceae* and *Eubacteriaceae*. However, the families with negative LFC with mg/kg, *Paenibacillaceae* and *Coriobacteriales Incertae Sedis*, differed from those found for the DDD metrics. We again found similar results for mg/kg as DDD for bacterial genera, with *Corynebacterium*, *Clostridium sensu stricto 1*, *Shuttleworthia*, and *Turicibacter* having positive LFC and *Psuedobutyrivibrio* having negative LFC (Figure 6D). *Lachnospiraceae UCG-007* additionally increased with mg/kg, whereas *Moryella* and *Acetobacter* had negative LFC with increasing mg/kg.

## 4. Discussion

In this study, we sought to compare the fecal bacterial community compositions of cattle from large conventional dairy farms that had high and low antimicrobial use (AMU). Our analyses found that fecal bacterial communities of cattle differed between high- and low-AMU farms and that this relationship was consistent even when using two different AMU quantification metrics. For this work, we aimed to account for natural variation across farm environments while assessing AMU impacts by collecting samples from multiple cattle groups with varying antimicrobial exposures, management practices, and microbiome compositions across a farm. We additionally sought to account for longitudinal variations by sampling each cattle group over four timepoints and for animal-to-animal variation by randomly and systematically collecting samples from multiple individuals from each cattle group on each farm.

We found the fecal bacterial communities of dairy cattle to differ between all four cattle groups (preweaned calves, healthy lactating cows, cull cows, and sick cows) (Figure S4). Although exploring the differences in fecal microbiomes among cattle groups was outside the main scope of this study, our results align with prior work on dairy farms that corroborate the expected differences in fecal microbiomes of dairy calves and adult animals [43,44]. However, we note that to our knowledge, no prior studies have directly compared the fecal microbiomes of healthy, cull, and sick cows on dairy farms. The differences in the microbiomes of sick cows are not surprising, as these animals are often undergoing antimicrobial treatment, and numerous studies have demonstrated short-term shifts in the fecal microbiome in response to antimicrobial treatment [15,16,17,20]. The differences we observed between cull cows and healthy lactating cows may be a result of age, as multiparous cattle are often more likely to be culled [45], and recent work has shown that the microbiomes of cattle shift with parity [46]. Other factors that lead to culling include, but are not limited to, poor udder health, lameness, and reproductive issues. Ultimately, the reasons for culling are dependent on decisions made by farm management, and the role of these collective decisions on the bovine fecal microbiome are complex and warrant further study. Additionally, diet can vary among cattle groups and potentially impact fecal microbiomes. Although feed data were not collected for this study, cull and healthy cows were co-housed on all eight farms and likely fed the same diet, suggesting that factors beyond diet drove differences between healthy and cull cows. Further research is needed to assess the role of diet in differences observed in sick cow fecal microbiomes.

We found no significant variation in fecal microbiomes across the visits to each farm. While prior work has demonstrated differential pathogen shedding in response to seasonal variation [47,48,49], we did not see any differences across time points. One reason could be that only a few visits were conducted during winter months (January–March), with the remainder being collected during summer and early fall (June–October), before temperatures began to drop. Other studies that have considered temporal changes in both microbiomes of cow feces and their environment found seasonal shifts in environmental samples but not in the dairy cow feces [50]. These observations suggest that the internal temperature of animals may protect the fecal microbiome from seasonal shifts, even though recovery of enteric pathogens is still variable.

We also found cattle fecal microbiomes to vary significantly across both the high- and low-AMU farms. Farm-to-farm variability has been identified as a potentially confounding factor in previous studies that have sought to examine cattle fecal microbiomes and resistomes [14,51] and may be due to a variety of factors, including geographic location, bedding materials, diet, barn design, and variation in farm management practices. Here, we attempted to control for this variability by including individual farm in our statistical models while evaluating farm AMU effects.

Our study also compared two different standard AMU quantification methods within the context of the microbiome. Compared to the animal defined daily dose (DDD) method, the mg/kg method overemphasizes antimicrobials with lower dosing concentrations over those with high dosing concentrations in addition to injectable over intramammary antimicrobial use [25]. Moreover, orally administered boluses of antimicrobials, often used in calves, are more favored when using the mg/kg metric. Here, we found noticeable differences in the detectable taxa of microbiomes from calves using the mg/kg metric when compared to the DDD metric or high/low AMU, with the former identifying 17 bacterial families as differentially abundant, while the others only identified single taxonomic groups. However, we note that the majority of the 17 identified bacterial taxa using mg/kg only had low LFC per unit increases in mg/kg. Beyond these differences in differentially abundant taxa in calves, we found only minor differences between AMU quantification methods. This finding was reflected in our alpha diversity metrics, where we found that Chao’s richness differed with AMU for calves but not for cows and that Shannon’s diversity differed with AMU in cows but not in calves. This suggests that the number of unique organisms in calves change with AMU, but their overall distributions (evenness) within those communities does not, whereas for cows, the number of unique bacterial taxa does not change with AMU, but their evenness within those communities does change.

We found similar results when comparing bacterial beta diversity between high- and low-AMU farms or by DDD and mg/kg as continuous variables. We found that the fecal bacterial communities of all cows did not significantly differ (weighted UniFrac) by DDD and only trended towards significance for mg/kg. This agrees with our earlier findings suggesting that when taking phylogenetic relatedness of ASVs into account, the community compositions are distinct between low- and high-AMU farms only when ASV presence/absence is considered (unweighted UniFrac) but not when evenness of the populations is also considered (weighted UniFrac). For calves, we found a trending P-value with mg/kg as an AMU metric, which suggests that mg/kg may be a more representative metric for antimicrobial exposures in the calf gastrointestinal tract.

Of the organisms we identified as potentially driving the differences between high- and low-AMU farms, several were consistently detected regardless of the AMU metric used. The most prominent of these consistently detected taxa was the genus *Corynebacterium*, which had the largest LFC with increasing DDD and mg/kg metrics in cows as well as being significantly enriched in high-AMU vs low-AMU farms. Members of the genus *Corynebacterium* include opportunistic mastitis pathogens, most notably *C. bovis*, which is associated with both subclinical and clinical mastitis cases in dairy cows [52,53]. *C. bovis* has been frequently isolated from bovine milk [54,55] but not from the dairy environment, indicating that environmental reservoirs are likely not important for its dissemination [56]. However, other *Corynebacterium* spp. isolated from milk and teat skin are known to be recovered from the dairy farm environment [56]. While prior studies comparing *Corynebacterium* spp. abundances between farms with variable AMU are limited, prior efforts have found *Corynebacterium* spp. from dairy milk to have low amounts of antimicrobial resistance [57,58] and to be less abundant in the milk and colostrum of dairy cattle previously treated with antimicrobials at dry-off than in untreated cows [59]. Taken together, these findings suggest that the increase in *Corynebacterium* abundance with increased AMU in our study is not likely due to direct selection in response to antimicrobial exposure and may be due to other underlying microbial community dynamics that require additional study to further elucidate.

In calves, for DDD, we only found the genus *Fusobacterium* to be negatively associated with increasing DDD. This suggests that increasing antimicrobial use, as measured by DDD, reduces the prevalence of this genus. *Fusobacterium* spp. have been previously found to be significantly more abundant in diarrheic calves than in non-diarrheic calves [60] and have been reported to be associated with the protozoal parasite *Cryptosporidium parvum*, a causative agent of diarrhea in calves [61]. Prior work examining the fecal microbiomes of calves fed waste milk containing antimicrobial residues found lower abundances of *Fusobacterium* when compared to calves fed bulk tank milk [62]. Additionally, *Fusobacterium* isolates from dairy milk were previously found to be susceptible to penicillin G [63], which constituted the highest proportion (~32%) of total preweaned calf AMU from the 40 dairy farms initially surveyed using the DDD metric and fourth highest proportion (~9%) using mg/kg [25], potentially explaining the reduction of *Fusobacterium* in calves on farms with higher AMU.

In this study, we used quantitative data on AMU of large conventional dairy farms in Wisconsin to better understand the bacterial dynamics of cattle feces in response to farm AMU. We unexpectedly found differences in the fecal microbiomes of cattle from high- and low-AMU farms, but we also found high farm-to-farm variability across fecal microbiomes. Of those significant differences, farm AMU only accounted for ~2.5% of the variance when comparing bacterial community compositions at the herd level and 6–11% of variance when comparing AMU metrics of calves and cows separately, indicating that AMU alone is only contributing a small percentage of the total variation of the fecal bacterial communities between herds. Although we attempted to account for variation among cattle groups and farms, this work was still constrained by several additional factors, the most notable of which was the time between AMU data collection in 2017 and microbiome sampling in 2020. Though subsequent sampling would have been preferred, farm AMU was not expected to differ during that period, as prior studies quantifying dairy farm AMU report consistent average AMU across dairy farms regardless of size, year surveyed (2007–2021), location/country, or method of data collection [25,28,29,64,65,66]. Additionally, there were no major changes in regulations or drug availability regarding AMU on dairy farms in the USA during this period to prompt the large (~3x) change in the use practice needed to cause a farm to switch AMU groups in this study. Furthermore, we asked farm managers at sampling onset if they had changed their AMU practices since AMU quantification. All stated that they had not changed their AMU practices.

An additional limitation was that we sought to evaluate broad changes in the microbiome using randomized composite sampling and AMU data collected at the farm level. Though useful for our initial aims, these methods overlook animal-level variation in both microbiome and antimicrobial exposure that may further elucidate conditions where AMU is most likely to cause shifts in the microbiome. Lastly, we were only able to sample eight farms (four farms per AMU group), which was further compounded by only two of the low-AMU farms rearing calves on-site, reducing our ability to detect variations in the microbiome between high- and low-AMU farms. As such, future studies should aim to increase the number of high- and low-AMU farms sampled to reduce the impacts of farm-to-farm variability on their results.

Despite these limitations, we found that the fecal microbiome of dairy cattle differed with AMU and further identified several taxonomic groups differentially abundant with farm AMU. Future work should consider this interplay and how AMU may translate to ARG emergence and transmission on conventional dairy farms, particularly as it relates to pathogens.

## 5. Conclusions

We found that bacterial community compositions of cattle feces differ between farms with high and low AMU but found AMU to only explain ~2.5% of the total microbiome variance across all samples, indicating that AMU alone explains a small percentage of the total variation of fecal bacterial communities across herds. We further discovered that the use of different AMU quantification metrics can impact a farm’s AMU ranking but does not change microbiome conclusions at the herd level. Despite substantial variability among farms and sampling a relatively small number of farms, we were able to identify bacterial taxa that may be selected by dairy farm AMU practices, including several potential pathogens that warrant additional investigation. This study furthers our understanding of the influence dairy farm AMU has on the dairy cattle fecal microbiome and provides a framework for future work to better understand antimicrobial resistance emergence and dissemination on dairy farms.

## Data Availability

The sequencing datasets generated and analyzed during the current study are available in the NCBI Sequence Read Archive repository under BioProject number PRJNA1093066 [https://www.ncbi.nlm.nih.gov/bioproject/PRJNA1093066 (accessed on 5 June 2025)].

## Supplementary Materials

The following supporting information can be downloaded at https://www.mdpi.com/article/10.3390/ani15121735/s1, Figure S1: Bacterial community compositions of each cattle group across enrolled farms; Figure S2: Diversity of calf and cow samples across high-AMU farms; Figure S3: Diversity of calf and cow samples across low-AMU farms; Figure S4: Comparison of bacterial diversity between cattle groups; Table S1: Sample metadata; Table S2: Sampling results.

## Abbreviations

The following abbreviations are used in this manuscript:

AMU	Antimicrobial use
DDD	Animal defined daily dose
ARG	Antimicrobial resistance gene
LFC	Log-fold change
KW	Kruskal–Wallis
