# Supplementary material for: Assessing the Impacts of Dairy Farm Antimicrobial Use on the Bovine Fecal Microbiome
Source: Animals (Basel). 2025 Jun 12;15(12):1735. doi: 10.3390/ani15121735 (PMC12189718; doi:10.3390/ani15121735)
Supplement: Supplementary file 1 [file animals-15-01735-s001.zip › Supplementary Figures.pdf]

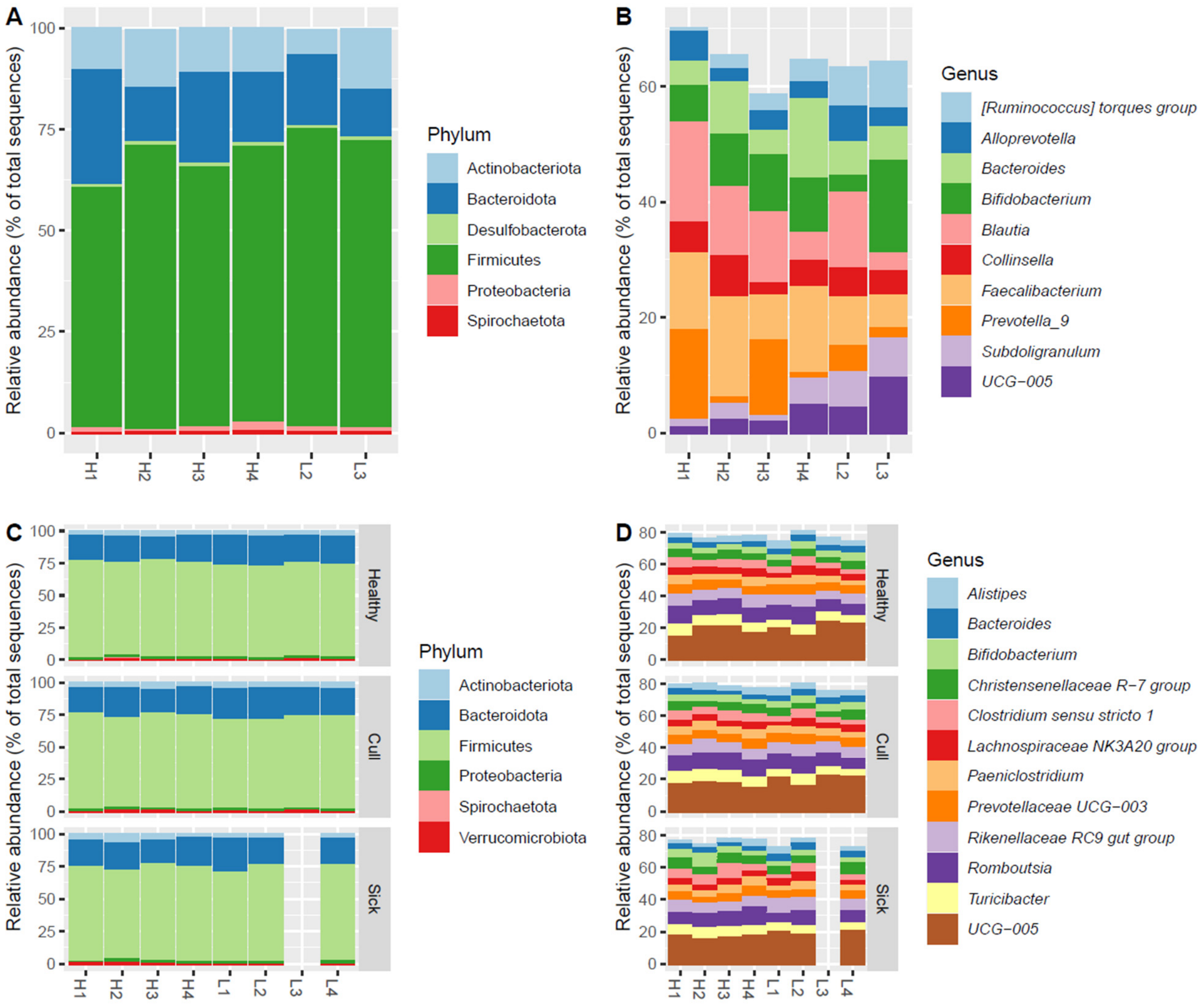


**Figure S1.** Bacterial community compositions of each cattle group across enrolled farms. Bar plots of the bacterial taxonomic compositions at the phylum and genus level of feces from preweaned calves (A, B), and cull, sick, and healthy lactating cows (C, D). Results are averaged across the 4 samples collected from each cattle group per farm. Only the 6 most abundant Phyla are reported and the 10 most abundant genera for calves, and 12 most abundant genera for cows are reported. Calf feces were not collected from farms L1 and L4 and are excluded from A and B. Farm L3 did not have a sick pen resulting in the blank bars in C and D.

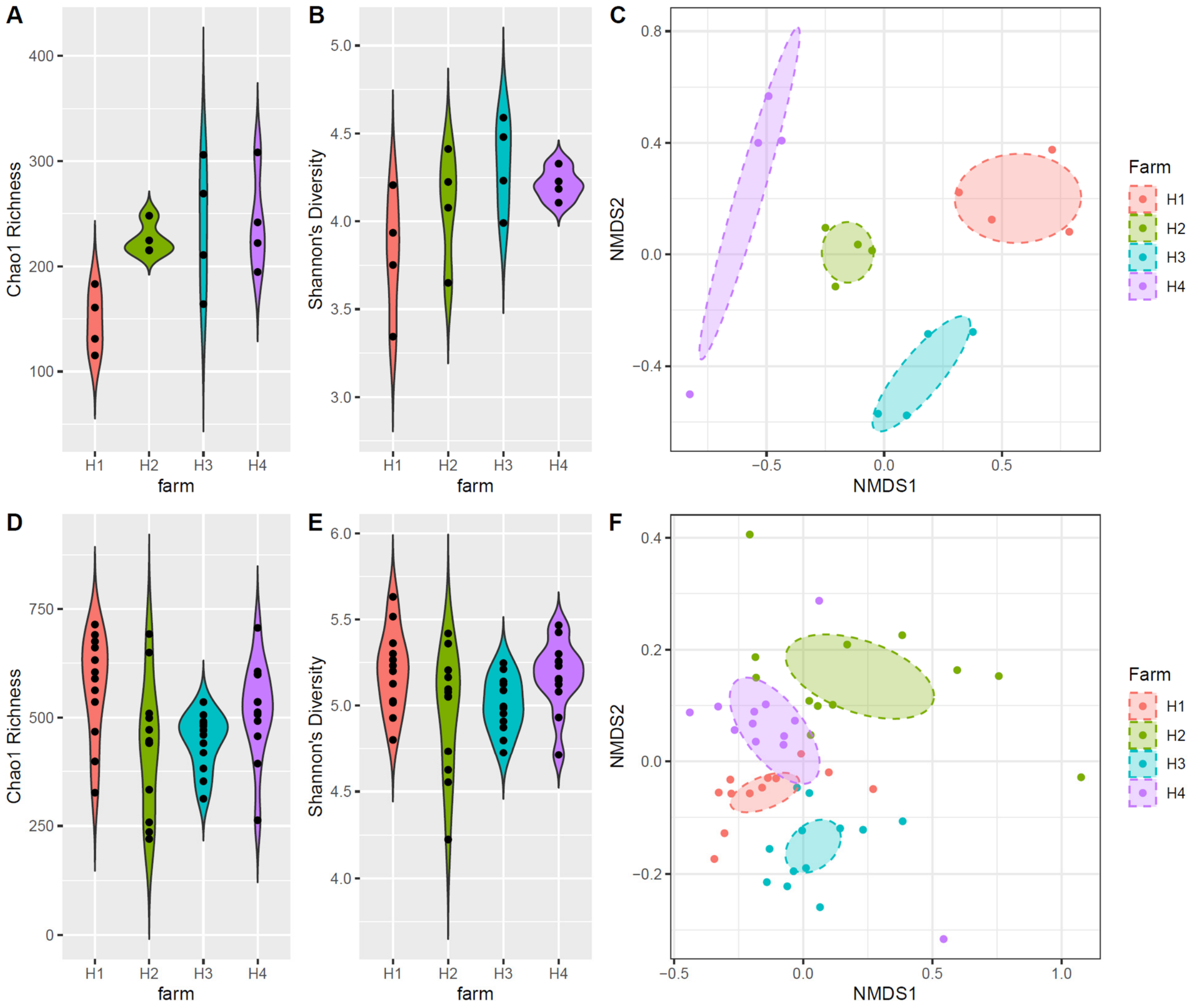


**Figure S2.** Diversity of calf and cow samples across high AMU farms. Violin plots comparing the Chao1 richness and Shannon's diversity of calf (A, B) and cow (D, E) fecal samples between the high AMU farms. Chao differed between farms for calves (p-value = 0.03) and cows (p-value = 0.02) while Shannon did not for calves (p-value = 0.11) but trended for cows (p-value = 0.058). Plots C and F are nMDS plots of the Bray-Curtis dissimilarity distances between fecal bacterial communities of calves (C) and cows (F) from high AMU farms. The bacterial community compositions differed between all high AMU farms for both calf and cow samples (P-value < 0.001). Stress: C = 0.1, F = 0.13.

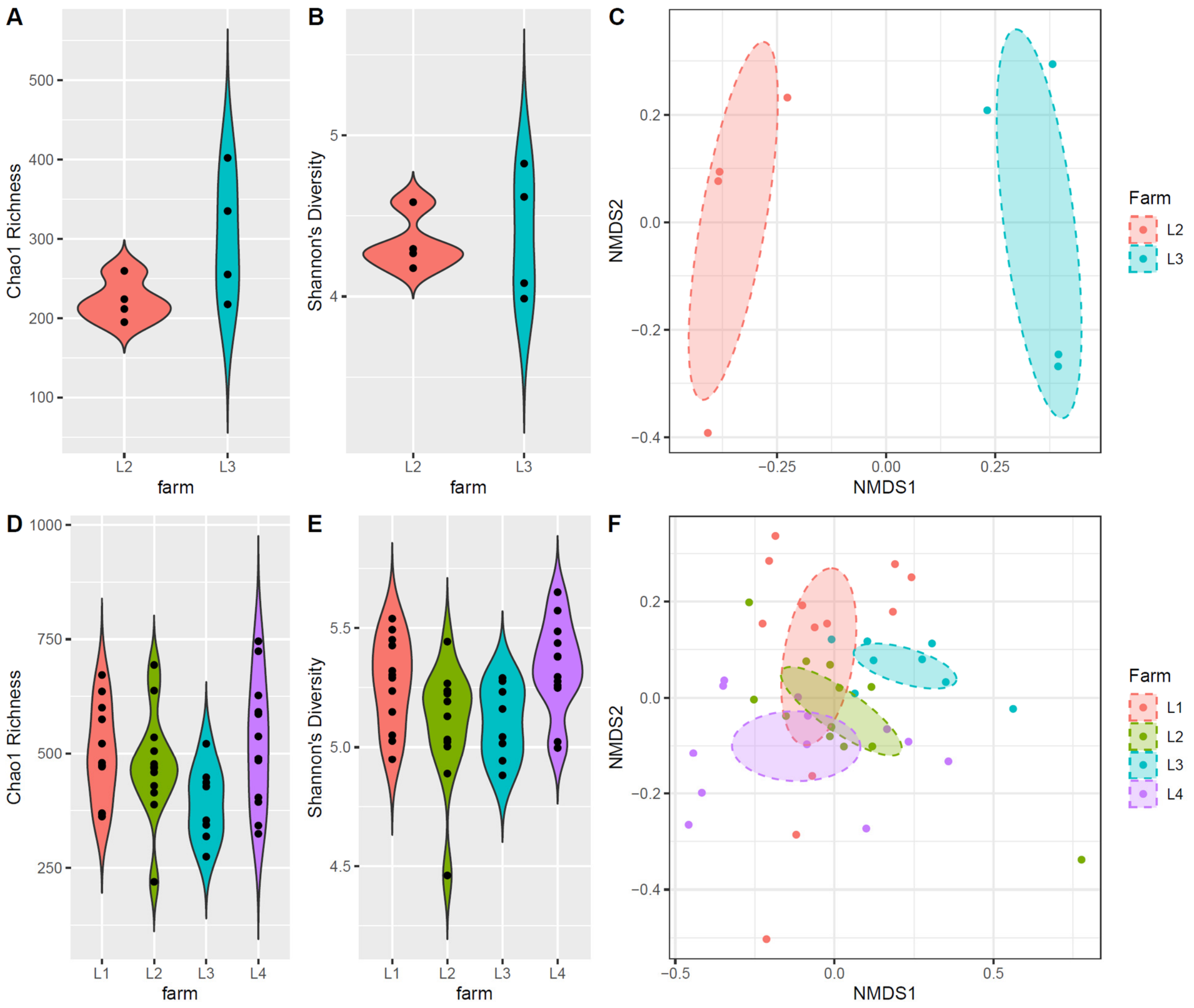


**Figure S3.** Diversity of calf and cow samples across low AMU farms. Violin plots comparing the Chao1 richness and Shannon's diversity of calf (A, B) and cow (D, E) fecal samples between the low AMU farms. Chao did not differ between farms for calves (p-value = 0.12) or cows (p-value = 0.1). Shannon also did not differ between low AMU farms for calves (p-value = 0.84) but did for cows (p-value = 0.02). Plots C and F are nMDS plots of the Bray-Curtis dissimilarity distances between fecal bacterial communities of calves (C) and cows (F) from low AMU farms. The bacterial community compositions differed between all low AMU farms for both calf (P-value = 0.03) and cow samples (P-value < 0.001). Stress: C = 0.04, F = 0.15.

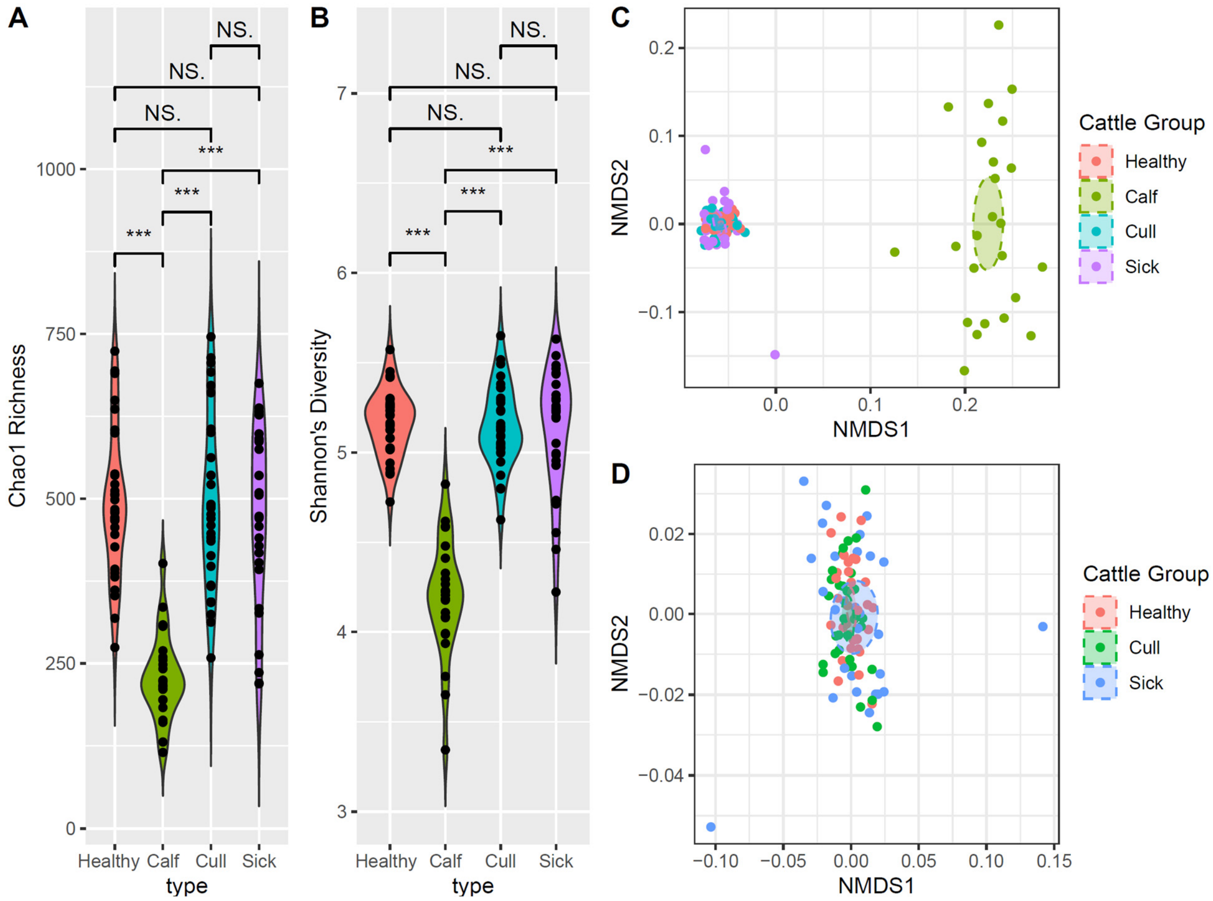


**Figure S4.** Comparison of bacterial diversity between cattle groups. Cattle groups differences by alpha diversity (A, B) and beta diversity (C). No differences were found for Chao richness (A) or Shannon's diversity (B) between adult cattle groups and calves had lower values for both metrics ($p$-value < 0.05). Beta-diversity was evaluated using weighted UniFrac distances and found the bacterial communities of each cattle group to be distinct (C) ($p$-value < 0.05 for each pairwise comparison). For better visualization of adult cattle groups, the NMDS was subset to only the cow groups in plot (D). Stress: C = 0.06, D = 0.13.
